# Supplementary material for: Navigating directed evolution efficiently: optimizing selection conditions and selection output analysis
Source: Front Mol Biosci. 2024 Oct 8;11:1439259. doi: 10.3389/fmolb.2024.1439259 (PMC11493728; doi:10.3389/fmolb.2024.1439259)
Supplement: Supplementary file 1 [file DataSheet1.zip › S1.docx]

Supplementary Material


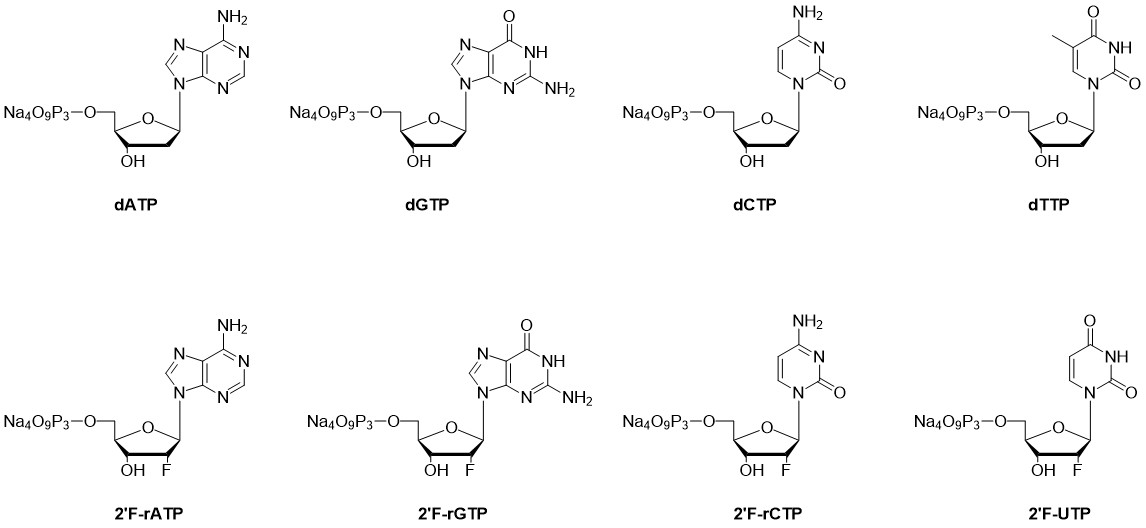


**Figure S1.** Chemical structures of nucleotide triphosphates used in this work.

| **Category** | **Name** | **Sequence** |
| --- | --- | --- |
| **Library construction** | KOD_Sat_403-404_F1 | CATAGTGTACNNKNNKTTTAGATCCCTGTACC |
|  | KOD_Sat_403-404_R1 | TTCTCCCACAACCCTCTC |
|  | KOD_5ptSat_408-409_F1 | AGATCCNNKNNKCCCTCAATCATC |
|  | KOD_5ptSat_403-405_R1 | KNNKNNKNNGTACACTATGTTCTCCCAC |
|  | PH_Delta_KOD_F1 | TCCCTGTACCCCTCAATC |
|  | PH_Delta_KOD_R1 | ATCTTCCATCGAGTAGCG |
| **Selection** | CSR_Sel_SHORT_F3 | cgagctgatcatcaacGAAATAACCACAGCCTGG |
|  | CSR_Sel_SHORT_R3 | cagctcatgctatatctCCGTTACGCTCTCTGC |
|  | CSR_Rec_SHORT_F1 | CGAGCTGATCATCAAC |
|  | CSR_Rec_SHORT_R1 | CAGCTCATGCTATATCC |
|  | spCSR_Innest_MUT_F3 | aaaGCTCTTCtGAAGGTCACATAtGAGCTTGGGAAG |
|  | spCSR_Innest_MUT_R3 | aaaGCTCTTCtCCTTGCATAcCCGTAGTAACCGTAG |
| **NGS Amplicon generation** | WP2_D1_Seq_F1_R0 | atgGCTACTCGATGGAAGATG |
|  | WP2_D1_Seq_F1_lib2 | atgGCTACTCGATGGAAGATG |
|  | WP2_D1_Seq_F1_lib5 | tgaGCTACTCGATGGAAGATG |
|  | WP2_D1_Seq_F1_lib7 | gatGCTACTCGATGGAAGATG |
|  | WP2_D1_Seq_F1_lib8 | actGCTACTCGATGGAAGATG |
|  | WP2_D1_Seq_F1_lib11 | cagGCTACTCGATGGAAGATG |
|  | WP2_D1_Seq_R1 | CCTCTAGGAGGTCTCCTAG |
|  | WP2_D4_Seq_F1 | gatGCTACTCGATGGAAGATG |
|  | WP2_D4_Seq_R1 | CCTCTAGGAGGTCTCCTAG |
| **Generation of polymerase variants for PCR** | KOD_D404S_fw | ACATAGTGTACCTATCTTTTAGATCC |
|  | KOD_L403F_fw | ACATAGTGTACTTCGATTTTAGATCC |
|  | KOD_L403V-D404S_fw | ACATAGTGTACGTATCTTTTAGATCC |
|  | KOD_L403V_fw | ACATAGTGTACGTAGATTTTAGATCC |
|  | KOD_mut_rv1 | TCTCCCACAACCCTC |
|  | KOD_Y409S_H5 | AGATCCCTGTCTCCCTCAATCATCATC |
|  | KOD_mut_rv3 | AAAATCTAGGTACACTATG |

**Table S1.** Sequences of the oligonucleotides and templates used in this study. All sequences are written in the 5’🡪3’ direction. N:A/C/G/T; K:G/T

| **pET23-KOD-Exo-**  TGGCGAATGGGACGCGCCCTGTAGCGGCGCATTAAGCGCGGCcgcTGTGGTGGTTACGCGCAGCGTGACCGCTACACTTGCCAGCGCCCTAGCGCCCGCTCCTTTCGCTTTCTTCCCTTCCTTTCTCGCCACGTTCGCCGGCTTTCCCCGTCAAGCTCTAAATCGGGGGCTCCCTTTAGGGTTCCGATTTAGTGCTTTACGGCACCTCGACCCCAAAAAACTTGATTAGGGTGATGGTTCACGTAGTGGGCCATCGCCCTGATAGACGGTTTTTCGCCCTTTGACGTTGGAGTCCACGTTCTTTAATAGTGGACTCTTGTTCCAAACTGGAACAACACTCAACCCTATCTCGGTCTATTCTTTTGATTTATAAGGGATTTTGCCGATTTCGGCCTATTGGTTAAAAAATGAGCTGATTTAACAAAAATTTAACGCGAATTTTAACAAAATATTAACGTTTACAATTTCAGGTGGCACTTTTCGGGGAAATGTGCGCGGAACCCCTATTTGTTTATTTTTCTAAATACATTCAAATATGTATCCGCTCATGAGACAATAACCCTGATAAATGCTTCAATAATATTGAAAAAGGAAGAGTATGAGTATTCAACATTTCCGTGTCGCCCTTATTCCCTTTTTTGCGGCATTTTGCCTTCCTGTTTTTGCTCACCCAGAAACGCTGGTGAAAGTAAAAGATGCTGAAGATCAGTTGGGTGCACGAGTGGGTTACATCGAACTGGATCTCAACAGCGGTAAGATCCTTGAGAGTTTTCGCCCCGAAGAACGTTTTCCAATGATGAGCACTTTTAAAGTTCTGCTATGTGGCGCGGTATTATCCCGTATTGACGCCGGGCAAGAGCAACTCGGTCGCCGCATACACTATTCTCAGAATGACTTGGTTGAGTACTCACCAGTCACAGAAAAGCATCTTACGGATGGCATGACAGTAAGAGAATTATGCAGTGCTGCCATAACCATGAGTGATAACACTGCGGCCAACTTACTTCTGACAACGATCGGAGGACCGAAGGAGCTAACCGCTTTTTTGCACAACATGGGGGATCATGTAACTCGCCTTGATCGTTGGGAACCGGAGCTGAATGAAGCCATACCAAACGACGAGCGTGACACCACGATGCCTGCAGCAATGGCAACAACGTTGCGCAAACTATTAACTGGCGAACTACTTACTCTAGCTTCCCGGCAACAATTAATAGACTGGATGGAGGCGGATAAAGTTGCAGGACCACTTCTGCGCTCGGCCCTTCCGGCTGGCTGGTTTATTGCTGATAAATCTGGAGCCGGTGAGCGTGGGTCTCGCGGTATCATTGCAGCACTGGGGCCAGATGGTAAGCCCTCCCGTATCGTAGTTATCTACACGACGGGGAGTCAGGCAACTATGGATGAACGAAATAGACAGATCGCTGAGATAGGTGCCTCACTGATTAAGCATTGGTAACTGTCAGACCAAGTTTACTCATATATACTTTAGATTGATTTAAAACTTCATTTTTAATTTAAAAGGATCTAGGTGAAGATCCTTTTTGATAATCTCATGACCAAAATCCCTTAACGTGAGTTTTCGTTCCACTGAGCGTCAGACCCCGTAGAAAAGATCAAAGGATCTTCTTGAGATCCTTTTTTTCTGCGCGTAATCTGCTGCTTGCAAACAAAAAAACCACCGCTACCAGCGGTGGTTTGTTTGCCGGATCAAGAGCTACCAACTCTTTTTCCGAAGGTAACTGGCTTCAGCAGAGCGCAGATACCAAATACTGTaCTTCTAGTGTAGCCGTAGTTAGGCCACCACTTCAAGAACTCTGTAGCACCGCCTACATACCTCGCTCTGCTAATCCTGTTACCAGTGGCTGCTGCCAGTGGCGATAAGTCGTGTCTTACCGGGTTGGACTCAAGACGATAGTTACCGGATAAGGCGCAGCGGTCGGGCTGAACGGGGGGTTCGTGCACACAGCCCAGCTTGGAGCGAACGACCTACACCGAACTGAGATACCTACAGCGTGAGCTATGAGAAAGCGCCACGCTTCCCGAAGGGAGAAAGGCGGACAGGTATCCGGTAAGCGGCAGGGTCGGAACAGGAGAGCGCACGAGGGAGCTTCCAGGGGGAAACGCCTGGTATCTTTATAGTCCTGTCGGGTTTCGCCACCTCTGACTTGAGCGTCGATTTTTGTGATGCTCGTCAGGGGGGCGGAGCCTATGGAAAAACGCCAGCAACGCGGCCTTTTTACGGTTCCTGGCCTTTTGCTGCGTTATCCCCTGATTCTGTGGCCTTTTGCgCgtCTGCGTTATCCCCTGATTCTGatgttctttcCTGCGTTATCCCCTGATTCTGtggataaccgtattaccgcctttgagtgagCTGCGTTATCCCCTGATTCTGCTGATACCGCTCGCCGCAGCCGAACGACCGAGCGCAGCGAGTCAGTGAGCGAGGAAGCGGAAtAtCGCCTGATGCGGTATTTTCTCCTTACGCATCTGTGCGGTATTTCACACCGCAATGGTGCACTCTCAGTACAATCTGCTCTGATGCCGCATAGTTAAGCCAGTATACACTCCGCTATCGCTACGTGACTGGGTCATGGCTGCGCCCCGACACCCGCCAACACCCGCTGACGCGCCCTGACGGGCTTGTCTGCTCCCGGCATCCGCTTACAGACAAGCTGTGACCGTCTCCGGGAGCTGCATGTGTCAGAGGTTTTCACCGTCATCACCGAAACGCGCGAGGCAGCTGCGGTAAAGCTCATCAGCGTGGTCGTGAAGCGATTCACAGATGTCTGCCTGTTCATCCGCGTCCAGCTCGTTGAGTTTCTCCAGAAGCGTTAATGTCTGGCTTCTGATAAAGCGGGCCATGTTAAGGGCGGTTTTTTCCTGTTTGGTCACTGATGCCTCCGTGTAAGGGGGATTTCTGTTCATGGGGGTAATGATACCGATGAAACGAGAGAGGATGCTCACGATACGGGTTACTGATGATGAACATGCCCGGTTACTGGAACGTTGTGAGGGTAAACAACTGGCGGTATGGATGCGGCGGGACCAGAGAAAAATCACTCAGGGTCAATGCCAGCGCTTCGTTAATACAGATGTAGGTGTTCCACAGGGTAGCCAGCAGCATATGGTGCAGGGCGCTGACTTCCGCGTTTCCAGACTTTACGAAACACGGAAACCGAAGACCATTCATGTTGTTGCTCAGGTCGCAGACGTTTTGCAGCAGCAGTCGCTTCACGTTCGCTCGCGTATCGGTGATTCATTCTGCTAACCAGTAAGGCAACCCCGCCAGCCTAGCCGGGTCCTCAACGACAGGAGCACGATCATGCGCACCCGTGGCCAGGACCCAACGCTGCCCGAGATCTCGATCCCGCGAAATTAATACGACTCACTATAGGGAGACCACAACGGTTTCCCTCTAGAAATAATTTTGTTTAACTTTAAGAAGGAGATATACCATGGATCCTCTAGAGTCGACCTGCAGGCATGCAAGCTTGCGGCcacacAGGAGATAGTCATACATGAAACACAAAGAGGAGAAATTAACTATGAGAGGATCTCACCATCACCATCACCATACGGATCCAAGCGGCCTGGTGCCGCGCGGCAGCATGATCCTCGACACTGACTACATAACCGAGGATGGAAAGCCTGTCATAAGAATTTTCAAGAAGGAAAACGGCGAGTTTAAGATTGAGTACGACCGGACTTTTGAACCCTACTTCTACGCCCTCCTGAAGGACGATTCTGCCATTGAGGAAGTCAAGAAGATAACCGCCGAGAGGCACGGGACGGTTGTAACGGTTAAGCGGGTTGAAAAGGTTCAGAAGAAGTTCCTaGGGAGACCAGTTGAGGTCTGGAAACTCTACTTTACTCATCCGCAGGACGaaCCAGCGATAAGGGACAAGATACGAGAGCATCCAGCAGTTATTGACATCTACGAGTACGACATACCCTTCGCCAAGCGCTACCTCATAGACAAGGGATTAGTGCCAATGGAAGGCGACGAGGAGCTGAAAATGCTCGCCTTCGCGATTGCGACTCTCTACCATGAGGGCGAGGAGTTCGCCGAGGGGCCAATCCTTATGATAAGCTACGCCGACGAGGAAGGGGCCAGGGTGATAACTTGGAAGAACGTGGATCTCCCCTACGTTGACGTCGTCTCGACGGAGAGGGAGATGATAAAGCGCTTCCTCCGTGTTGTGAAGGAGAAAGACCCGGACGTTCTCATAACCTACAACGGCGACAACTTCGACTTCGCCTATCTGAAAAAGCGCTGTGAAAAGCTCGGAATAAACTTCGCCCTCGGAAGGGATGGAAGCGAGCCGAAGATTCAGAGGATGGGCGACAGGTTTGCCGTCGAAGTGAAGGGACGGATACACTTCGATCTCTATCCTGTGATAAGACGGACGATAAACCTGCCCACATACACGCTTGAGGCCGTTTATGAAGCCGTCTTCGGTCAGCCGAAGGAGAAGGTTTACGCTGAGGAAATAACCACAGCCTGGGAAACCGGCGAGAACCTTGAGAGAGTCGCCCGCTACTCGATGGAAGATGCGAAGGTCACATACGAGCTTGGGAAGGAGTTCCTTCCGATGGAGGCCCAGCTTTCTCGCTTAATCGGCCAGTCCCTCTGGGACGTCTCCCGCTCCAGCACTGGCAACCTCGTTGAGTGGTTCCTCCTCAGGAAGGCCTATGAGAGGAATGAGCTGGCCCCGAACAAGCCCGATGAAAAGGAGCTGGCCAGAAGACGGCAGAGCTATGAAGGAGGCTATGTAAAAGAGCCCGAGAGAGGGTTGTGGGAGAACATAGTGTACCTAGATTTTAGATCCCTGTACCCCTCAATCATCATCACCCACAACGTCTCGCCGGATACGCTCAACAGAGAAGGATGCAAGGAATATGACGTTGCCCCACAGGTCGGCCACCGCTTCTGCAAGGACTTCCCAGGATTTATCCCGAGCCTGCTaGGAGACCTCCTAGAGGAGAGGCAGAAGATAAAGAAGAAGATGAAGGCCACGATTGACCCGATCGAGAGGAAGCTCCTCGATTACAGGCAGAGGGCAATCAAGATCCTGGCAAACAGCTACTACGGTTACTACGGCTATGCAAGGGCGCGCTGGTACTGCAAGGAGTGTGCAGAGAGCGTAACGGCCTGGGGAAGGGAGTACATAACGATGACCATCAAGGAGATAGAGGAAAAGTACGGCTTTAAGGTAATCTACAGCGACACCGACGGATTTTTTGCCACAATACCTGGAGCCGATGCTGAAACCGTCAAAAAGAAGGCTATGGAGTTCCTCAAGTATATCAACGCCAAACTTCCGGGCGCGCTTGAGCTCGAGTACGAGGGCTTCTACAAACGCGGCTTCTTCGTCACGAAGAAGAAGTATGCGGTGATAGACGAGGAAGGCAAGATAACAACGCGCGGACTTGAGATTGTGAGGCGTGACTGGAGCGAGATAGCGAAAGAGACGCAGGCGAGGGTTCTTGAAGCTTTGCTAAAGGACGGTGACGTCGAGAAGGCCGTGAGGATAGTCAAAGAAGTTACCGAAAAGCTGAGCAAGTACGAGGTTCCGCCGGAGAAGCTGGTGATCCACGAGCAGATAACGAGGGATTTAAAGGACTACAAGGCAACCGGTCCCCACGTTGCCGTTGCCAAGAGGTTGGCCGCGAGAGGAGTCAAAATACGCCCTGGAACGGTGATAAGCTACATCGTGCTCAAGGGCTCTGGGAGGATAGGCGACAGGGCGATACCGTTCGACGAGTTCGACCCGACGAAGCACAAGTACGACGCCGAGTACTACATTGAGAACCAGGTTCTCCCAGCCGTTGAGAGAATTCTGAGAGCCTTCGGTTACCGCAAGGAAGACCTGCGCTACCAGAAGACGAGACAGGTTGGTTTGAGTGCTTGGCTGAAGCCGAAGGGAACTTGATCGATGCTCCGAGATGAGGTAGGATGGCTGGCTTACGGTGTTACTGCTGAGGAATGAgccatcCTCGAGCACCACCACCACCACCACTGAGATCCGGCTGCTAACAAAGCCCGAAAGGAAGCTGAGTTGGCTGCTGCCACCGCTGAGCAATAACTAGCATAACCCCTTGGGGCCTCTAAACGGGTCTTGAGGGGTTTTTTGCTGAAAGGAGGAACTATATCCGGAT |
| --- |

**Table S2.** Sequences of all the plasmids used in this study.

| **Pipeline step** | **Sequences output R0** | **Sequences output R1** | | | | |
| --- | --- | --- | --- | --- | --- | --- |
|  |  | **Sel 2** | **Sel 5** | **Sel 7** | **Sel 8** | **Sel 11** |
| **Total reads** | 466,836 | 462,439 | | | | |
| **Total paired reads** | 433794 (93%) | 374,592 (81%) | | | | |
| **Quality filtering** | 417398 (89%) | 355,811 (77%) | | | | |
| **Filtering by 3’ and 5’ sequence and translating** | 211,965* (45%) | 55,228* (12%) | 29,657* (6%) | 28,297* (6%) | 42,943* (9%) | 64,898* (14%) |
| **Unique sequences** | 450 | 338 | 310 | 295 | 314 | 335 |
| **Coverage** | 530x | 138x | 74x | 71x | 107x | 162x |

**Table S3.** Analysis by next generation sequencing of the Design 1 recovered **sequences.** Total read number obtained and the impact of the analysis pipeline are shown. *Number of sequences used in downstream analysis.

| **Pipeline step** | **Sequences output R0** | **Sequences output R1** | | |
| --- | --- | --- | --- | --- |
|  |  | **Sel 4** | **Sel 8** | **Sel 20** |
| **Total reads** | 2,508,732 | 753,984 | 911,540 | 833,199 |
| **Total paired reads** | 2,128,712 (85%) | 695,596 (92%) | 830,478 (91%) | 790,840 (95%) |
| **Quality filtering** | 2,030,557 (81%) | 667,972 (89%) | 790,840 (87%) | 689,774 (83%) |
| **Filtering by 3’ and 5’ sequence and translating** | 1,400,528 (56%) | 577,132 (77%) | 668,635 (73%) | 566,024 (68%) |
| **Unique sequences** | 531,097 | 1,428 | 1,058 | 16,300 |
| **Coverage** | 0.5x | 0.2x | 0.2x | 0.2x |

**Table S4.** Analysis by next generation sequencing of the Design 4 recovered sequences. Total read number obtained and the impact of the analysis pipeline are shown. *Number of sequences used in downstream analysis.

| **D1: Innest28S** | | | | | | | | | | | | | | | |
| --- | --- | --- | --- | --- | --- | --- | --- | --- | --- | --- | --- | --- | --- | --- | --- |
|  | **BSA** | | **Betaine** | | **MgCl_2_** | **MnCl_2_** | | **PEG**  **1000** | | **Formamide** | | **NTP**  **conc.** | **Time** | | **2'F-rATP** |
| **Avg.** | 2.2x10^-1^ | | 2.4x10^-1^ | | 1.1x10^-1^ | 1.8x10^-1^ | | 3.0x10^-3^ | | 2.9x10^-1^ | | 7.7x10^-1^ | 1.0x10^-1^ | | 3.0x10^-1^ |
| **STD** | 4.6x10^-2^ | | 5.0x10^-2^ | | 2.3x10^-2^ | 3.7x10^-2^ | | 4.8x10^-3^ | | 6.0x10^-2^ | | 6.3x10^-2^ | 2.2x10^-2^ | | 6.4x10^-2^ |
| **D1: Innest28G** | | | | | | | | | | | | | | | |
|  | **BSA** | | **Betaine** | | **MgCl_2_** | **MnCl_2_** | | **PEG**  **1000** | | **Formamide** | | **NTP**  **conc.** | **Time** | | **2'F-rATP** |
| **Avg.** | 0 | | 0 | | 0 | 0 | | 0 | | 6.9 x10^-3^ | | 5.2x10^-1^ | 0 | | 0 |
| **STD** | 0 | | 0 | | 0 | 0 | | 0 | | 9.9x10^-3^ | | 2.2x10^-2^ | 0 | | 0 |
| **D2: innest28G** | | | | | | | | | | | | | | | |
|  | | **Betaine** | | **MnCl_2_** | | | **Formamide** | | **2'F-rATP** | | **2'F-rATP/2'F-rCTP** | | | **dNTPs** | |
| **Avg.** | | 6.2x10^-2^ | | 2.0x10^-1^ | | | 5.0x10^-5^ | | 3.9x10^-2^ | | 5.3x10^-2^ | | | 1.9 | |
| **STD** | | 1.1x10^-2^ | | 1.3x10^-2^ | | | 3.8x10^-4^ | | 3.1x10^-2^ | | 1.8x10^-2^ | | | 3.0x10^-2^ | |
| **D2: innest28S** | | | | | | | | | | | | | | | |
|  | | **Betaine** | | **MnCl_2_** | | | **Formamide** | | **2'F-rATP** | | **2'F-rATP/2'F-rCTP** | | | **dNTPs** | |
| **Abs avg.** | | 3.3x10^-3^ | | 0 | | | 0 | | 0 | | 0 | | | 1.9 | |
| **STD** | | 5.2x10^-3^ | | 0 | | | 0 | | 0 | | 0 | | | 2.3x10^-2^ | |

**Table S5.** Absolute Lasso Regression coefficients from DoE-CSR selection products. The average relative coefficient values for each factor were computed from 100 runs of Lasso regression models for each design using Innest28G and Innest28S responses. For each factor, the coefficient values across the 100 model runs were averaged, and the standard deviation was calculated. The absolute value of each coefficient as a measure of factor importance is shown.

| **D2: innest28G quadratic and interaction model** | | | | | | | | |
| --- | --- | --- | --- | --- | --- | --- | --- | --- |
| **Avg.** | **Betaine** | **MnCl_2_** | **Formamide** | **2'F-rATP** | **2'F-rATP/**  **2'F-rCTP** | **2'F-rATP/**  **2'F-rUTP** | **dNTPs** | **2'F-rUTP** |
| **Betaine** | 2.3 x10^-1^ | 0 | 0 | 0 | 0 | 0 | 1.2x10^-1^ | 5.6x10^-2^ |
| **MnCl_2_** | 0 | 5.0x10^-2^ | 0 | 5.0x10^-1^ | 9.4x10^-2^ | 5.8x10^-3^ | 4.5x10^-1^ | 1.1x10^-1^ |
| **Formamide** | 0 | 0 | 6.7x10^-4^ | 1.1x10^-1^ | 0 | 8.3x10^-2^ | 1.1x10^-3^ | 0 |
| **2'F-rATP** | 0 | 5.0x10^-1^ | 1.1x10^-1^ | 0 | 0 | 0 | 0 | 0 |
| **2'F-rATP/**  **2'F-rCTP** | 0 | 9.4x10^-2^ | 0 | 0 | 0 | 0 | 0 | 0 |
| **2'F-rATP/**  **2'F-rTTP** | 0 | 5.8x10^-3^ | 8.3x10^-2^ | 0 | 0 | 0 | 0 | 0 |
| **dNTPs** | 1.2x10^-1^ | 4.5x10^-1^ | 1.1x10^-3^ | 0 | 0 | 0 | 0 | 0 |
| **2'F-rTTP** | 5.6x10^-2^ | 1.1x10^-1^ | 0 | 0 | 0 | 0 | 0 | 0 |
| **D2: innest28S quadratic and interaction model** | | | | | | | | |
| **Avg.** | **Betaine** | **MnCl_2_** | **Formamide** | **2'F-dA** | **2'F-dA/**  **2'F-dC** | **2'F-dA/**  **2'F-dT** | **dNTPs** | **2'F-dT** |
| **dNTPs** | 7.8x10^-2^ | 3.4x10^-2^ | 0 | 0 | 0 | 0 | 0 | 0 |

**Table S6.** Absolute quadratic and interaction Lasso Regression coefficients from D2 DoE-CSR selection products. The average relative coefficient values of the interaction and quadratic terms were computed from 100 runs of the Lasso regression model using Innest28G and Innest28S responses. For each factor, the absolute coefficient values across the 100 model runs were averaged and displayed in a 2D format.
